# Accuracy of Intraocular Lens Power Calculation in Cataract Surgery Combined with Trabeculectomy in Open Angle Glaucoma

**DOI:** 10.3390/jcm15103883

**Published:** 2026-05-18

**Authors:** Giulia Coco, Giulia Piccotti, Federica Genova, Lucrezia Leucci, Danilo Iannetta, Gloria Roberti, Carlo Nucci

**Affiliations:** 1 Ophthalmology Unit, Department of Clinical Sciences and Translational Medicine, University of Rome Tor Vergata, 00133 Rome, Italy; giulia.piccotti@libero.it (G.P.); leuccilucrezia@gmail.com (L.L.); 2 Department of Ophthalmology, Policlinico Casilino, 00169 Rome, Italy; federica.genova@hotmail.it; 3 Department of Sense organs, University of Rome La Sapienza, 00161 Rome, Italy; danilo.iannetta@uniroma1.it (D.I.); gloria.roberti@fondazionebietti.it (G.R.); 4 IRCCS-Fondazione Bietti, 00184 Rome, Italy; 5 Ophthalmology Unit, Department of Experimental Medicine, University of Rome Tor Vergata, 00133 Rome, Italy; nucci@med.uniroma2.it

**Keywords:** intraocular lens, IOL accuracy, IOL formulas, refractive outcomes, phacotrabeculectomy, cataract, glaucoma

## Abstract

**Background/Objectives**: To assess the accuracy of several intraocular lens power calculation formulas in phacotrabeculectomy for open angle glaucoma. **Methods**: Patients who underwent phacotrabeculectomy for open angle glaucoma were included. Refraction and biometry measurements were repeated at 3, 6 and ≥12 months. Prediction error (PE) and absolute error (AE) were calculated using the SRK/T, Holladay 1, Hoffer Q, Haigis, Kane, Emmetropia Verifying Optical (EVO) and Barrett Universal II formulas at ≥12 months, and their accuracy was compared using linear mixed-effects models accounting for repeated measurements within the same eye and inter-eye correlation. **Results**: Sixty eyes from 40 patients were included. The linear mixed-effects model showed a significant overall effect of formula on PE (χ^2^(6) = 119.14, *p* < 0.001). Most formulas showed a tendency toward a hyperopic refractive shift, whereas Haigis showed a negative PE. Based on estimated marginal mean AE, the formulas were ranked as follows: EVO (0.548 D), Barrett Universal II (0.551 D), Holladay and SRK/T (0.561 D), Haigis (0.572 D), Kane (0.577 D) and Hoffer Q (0.617 D). However, the AE did not significantly differ among the formulas (χ^2^(6) = 3.75, *p* = 0.711). The percentage of eyes within ± 1.00D of PE ranged from 81.7% to 90% across the formulas (*p* > 0.05). Significant axial length shortening, anterior chamber deepening and mean keratometry reduction were detected postoperatively at ≥12 months (*p* < 0.05). **Conclusions**: Despite postoperative ocular anatomic changes, all formulas showed acceptable refractive accuracy after phacotrabeculectomy. Although no significant difference in the AE was detected among the formulas, the PE differed significantly, with most formulas showing a tendency toward a hyperopic shift and Haigis showing a myopic shift. This inter-formula difference should be considered when selecting the refractive target, particularly when using formulas that tend toward hyperopic PE.

## 1. Introduction

Trabeculectomy has traditionally been considered a reference surgical procedure to reduce intraocular pressure (IOP) in open angle glaucoma (OAG) [1]. Due to the average age at which glaucoma surgery is generally deemed necessary, it often coexists with the presence of cataract, and trabeculectomy itself can be a risk factor for cataract formation [2]. At the same time, cataract surgery alone in the early stages of glaucoma has been associated with IOP reduction both in OAG and angle closure glaucoma (ACG) by deepening the anterior chamber (AC), thus resolving angle crowding, and by a possible mechanism of trabecular meshwork remodeling [3,4,5,6,7]. Consequently, when planning surgery in a patient with both glaucoma and cataract, the decision on whether to perform trabeculectomy first, followed by cataract surgery; cataract surgery alone; or combined phacotrabeculectomy is a matter of debate.

Phacotrabeculectomy is indicated in cases of no adequate IOP control with medical treatment, limited tolerance to medical treatment, risk of visual field damage worsening due to postoperative IOP spikes, or when the patient’s compliance is in doubt and the patient is also diagnosed with cataract [3,4,8].

Although some evidence suggests that trabeculectomy alone in pseudophakic eyes has a higher success rate with less frequent bleb needling [9], more recent data with 24 months follow-up seem in favor of the combined procedure [10].

One of the disadvantages of performing cataract and glaucoma surgery in the same procedure is the risk of postoperative prediction errors (PE) when calculating the power of the intraocular lens (IOL) to implant [9]. In fact, it is known that, following glaucoma surgery, variations in corneal curvature, anterior chamber depth (ACD) and axial length (AXL) may take place, determining changes in the actual refraction. Anatomical changes were reported up to 60 months postoperatively, subject to variation during the first postoperative months [11,12]. Specifically, biometric changes appeared more pronounced within the first three months and to persist to at least 6 months postoperatively [13,14].

So far, limited data is available on which type of IOL power calculation formula might perform better in eyes undergoing phacotrabeculectomy. Most studies only focused on conventional formulas, such as the SRK/T, Holladay, and Hoffer Q and, more recently, on the Barrett Universal II formula [15,16,17,18].

However, most of these studies either included a limited number of eyes or examined postoperative refraction sooner than 6 months postoperatively or included both OAG and ACG in which anatomic changes are expected to be different [12,19]. In addition, the performance of new-generation formulas such as Kane and Emmetropia Verifying Optical (EVO) has never been explored [20].

Therefore, the purpose of this study was to assess the accuracy of several IOL power calculation formulas for cataract surgery when combined with trabeculectomy in OAG patients at 12 months postoperatively, when further anatomical changes are likely to be absent. In addition, secondary outcomes were to detect ocular anatomic changes over time and to estimate their role in refraction prediction.

## 2. Materials and Methods

A retrospective study conducted at the University Hospital of Rome Tor Vergata on patients undergoing phacotrabeculectomy for glaucoma and cataract. Only patients with a diagnosis of primary open angle glaucoma (POAG) were included. Glaucoma diagnosis was made by a glaucoma specialist based on the presence of glaucomatous optic neuropathy and a glaucomatous visual field defect. POAG was defined by the presence of an anterior chamber open angle evaluated by gonioscopy and the absence of secondary forms of glaucoma. The exclusion criteria were age < 18 years old, previous corneal refractive surgery, ocular disease that may affect refractive errors except for glaucoma and cataract, IOL in the ciliary sulcus or scleral fixation of IOL, surgery-related complications potentially affecting refractive outcomes, bleb needling after phacotrabeculectomy and preoperative and/or postoperative visual acuity ≥ 1.0 logMAR. All the patients underwent complete preoperative ophthalmological examination including corrected distance visual acuity (CDVA), IOP measurement with Goldmann applanation tonometry (Haag-Streit, Köniz, Switzerland), gonioscopy, slit-lamp anterior segment and dilated fundus exam. Flat keratometry (K1), steep keratometry (K2), and mean keratometry (Km), ACD and AXL were measured with the partial coherence interferometer (PCI) IOLMaster 500 (Carl Zeiss Meditec, Jena, Germany). All the patients underwent phacotrabeculectomy with implantation of monofocal, acrylic, one-piece IOL. All the study procedures adhered to the tenets of the Declaration of Helsinki and the study was approved by the local Ethical Committee.

### 2.1. Surgical Procedure

Surgeries were performed with a 2-site approach under peribulbar anesthesia. A traction 6-0 prolene suture was placed on the superior cornea to achieve adequate exposure of the surgical site. Fornix-based conjunctival flap was created first and gentle cautery was used to achieve hemostasis of scleral vessels. 0.02% mitomycin C (MMC) was applied through sponges for 3 min in the filtration area. The area exposed to MMC was then thoroughly irrigated with 60 mL of balanced salt solution (BSS). A rectangular 3 mm (L) × 4 mm (W) partial-thickness scleral flap was created in the superior sclera, hinged at the limbus. At this point cataract surgery was performed via temporal approach through a 2.4 mm main incision. After IOL implantation, sclerotomy with localized removal of Schlemm’s canal and iridectomy was performed. The scleral flap was sutured with two 10-0 nylon releasable sutures and the conjunctiva was closed with 8-0 vicryl sutures. Intracameral antibiotic injection and subconjunctival injection of steroids and antibiotics were performed at the end of the surgery. All the patients received postoperative treatment with topical atropine, antibiotic and steroids.

### 2.2. Intraocular Lens Power Calculation

The predicted spherical equivalent (SE) was calculated using the following formulas: SRK/T, Holladay 1, Hoffer Q, Haigis, Kane, EVO and Barrett Universal II. The User Group for Laser Interference Biometry (ULIB) constants within the IOLMaster 500 software were used for the SRK/T, Holladay 1, Haigis and Hoffer Q, while the online free calculators were used for new-generation formulas, using their recommended constants. The optical lens constants were not optimized by our postoperative data since a large number of cases would have been required to substantially improve refractive outcomes. Postoperative refraction was performed by an experienced optometrist. The prediction error (PE) was calculated as the actual postoperative SE refraction minus the SE predicted refraction [21]. Positive PE values indicated a hyperopic refraction compared to the target refraction while negative PE values indicated a myopic refraction. The absolute error (AE) was defined as the PE absolute value. Mean PE and mean AE were calculated for each formula, together with the percentages of eyes with PE within ±0.25 D, ±0.50 D, ±1.00 D and ±2.00 D in each formula [22].

Most patients also underwent repeated measurements of keratometry, ACD and AXL at 3, 6 and ≥12 months after surgery. Postoperative changes of keratometry, ACD and AXL over time were evaluated when data were available.

### 2.3. Statistical Analysis

Differences in PE and AE among the seven formulas were assessed using linear mixed-effects models. The PE or AE was entered as the dependent variable, the formula was included as a categorical fixed factor, and random intercepts for patient and eye were included to account for repeated measurements within the same eye and inter-eye correlation. Estimated marginal means with 95% confidence intervals were reported for the PE and AE for each formula. The overall fixed effect of formula was tested, and when significant, pairwise comparisons between formulas were performed using estimated marginal means with Bonferroni adjustment for multiple comparisons. Fisher’s exact test was used to assess the differences among the formulas in the percentage of eyes within ±0.50 D and ±1.00 D of PE.

Formula-specific associations between the PE or AE and preoperative IOP, keratometry readings, ACD, AXL, and postoperative changes in these parameters were assessed using separate linear mixed-effects models for each formula, with the patient included as a random effect to account for inter-eye correlation.

Continuous variables are presented as mean ± standard deviation or as estimated marginal means with 95% confidence intervals, as appropriate. Categorical variables are presented as frequencies and percentages.

Postoperative changes in keratometry readings, ACD, and AXL were expressed as a percentage change from the baseline. Given the limited number of eyes with available data at 3 and 6 months, these intermediate time-points were analyzed descriptively, whereas formal statistical comparisons were limited to the baseline versus ≥12-month data using the paired Wilcoxon signed-rank test.

Sample size calculation to detect significant differences in mean AE between two formulas was performed using a two-sample means test. Considering a significance level of 0.05 with 80% power, an allocation ratio of 1, an effect size of 0.25 D and a mean AE of 0.59 ± 0.44 D based on a previous study reporting the mean AE at 12 months following phacotrabeculectomy using the SRK/T formula [23], 50 patients per group were required to detect a 0.25 D reduction in mean AE with a potentially better-performing formula. All the statistical analyses were performed using Stata 19.0 (StataCorp, College Station, TX, USA) and a *p*-value of less than 0.05 was considered statistically significant.

## 3. Results

Sixty eyes from 40 patients undergoing phacotrabeculectomy with monofocal IOL implantation were included in the analysis. Of the 102 eyes initially screened, 42 were excluded: 22 because of missing postoperative refractive data, 8 because of bleb needling, 2 because of sulcus IOL implantation, and 10 because of surgery-related complications, potentially affecting refractive outcomes. These included IOL capture (n = 2), IOL dislocation (n = 3), IOL instability (n = 1), marked postoperative hypotony requiring additional surgery (n = 1), mild corneal edema (n = 1), iris incarceration in the trabeculectomy site (n = 1) and cystoid macular edema (n = 1). The demographics and baseline clinical characteristics are summarized in Table 1.

The preoperative IOP was 20.6 ± 7.7 mmHg. Visual field damage, expressed as mean deviation, was classified as mild (>−6 dB) in 10.0% of eyes (6/60), moderate (≤−6 dB and >−12 dB) in 18.3% (11/60) and severe (≤−12 dB) in 71.7% (43/60). The Bausch & Lomb enVista MX60PL (Bausch + Lomb, Rochester, NY, USA) was implanted in 48.3% of cases, the Alcon AcrySof IQ SN60WF (Alcon Laboratories, Fort Worth, TX, USA) in 28.3% of cases, the Alcon AcrySof IQ AU00T0 in 3.3% and the Hoya iSert 251 (Hoya Surgical Optics, Singapore) in 20% of cases. At 12 ± 4 months of follow-up, the IOP was significantly reduced by 6.2 ± 7.5 mmHg (*p* < 0.0001) and the CDVA significantly improved by 0.1 ± 0.3 logMAR (*p* = 0.0136). The mean PE was positive with most formulas, suggesting a tendency toward a hyperopic refractive shift, whereas Haigis showed a negative mean PE.

The linear mixed-effects model showed a significant overall effect of formula on PE (χ^2^(6) = 119.14, *p* < 0.001). The estimated marginal mean PE ranged from −0.220 for Haigis to 0.195 for Barrett Universal II. Only Barrett Universal II and Haigis showed PE values significantly different from zero, with Barrett Universal II showing a positive PE (0.195; 95% CI, 0.008 to 0.383) and Haigis showing a negative PE (−0.220; 95% CI, −0.407 to −0.032). The 95% confidence intervals for EVO, Hoffer Q, Holladay 1, Kane, and SRK/T included zero. Bonferroni-adjusted pairwise comparisons showed significantly lower PE for Haigis compared with all the other formulas, and for Kane compared with Barrett Universal II. No other pairwise comparisons were significant.

Based on estimated marginal mean AE, the formulas ranked as follows: EVO (0.548 D), Barrett Universal II (0.551 D), Holladay and SRK/T (0.561 D), Haigis (0.572 D), Kane (0.577 D) and Hoffer Q (0.617 D). However, the linear mixed-effects model did not show a significant overall effect of formula on AE (χ^2^(6) = 3.75, *p* = 0.711), indicating that AE did not significantly differ among the seven formulas (Table 2).

**Figure 1:**
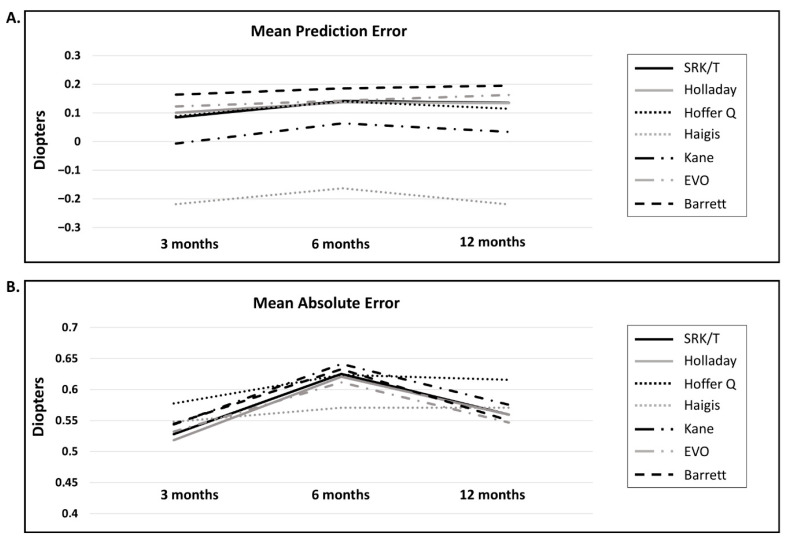
Mean prediction error and mean absolute error values at each time point with different biometric formulas (**A**) Mean Prediction Error; (**B**) Mean Absolute Error.

The postoperative trends of the mean PE and mean AE are shown descriptively in Figure 1. Most formulas showed no change in the sign of PE between 3 and 12 months postoperatively.

**Figure 2:**
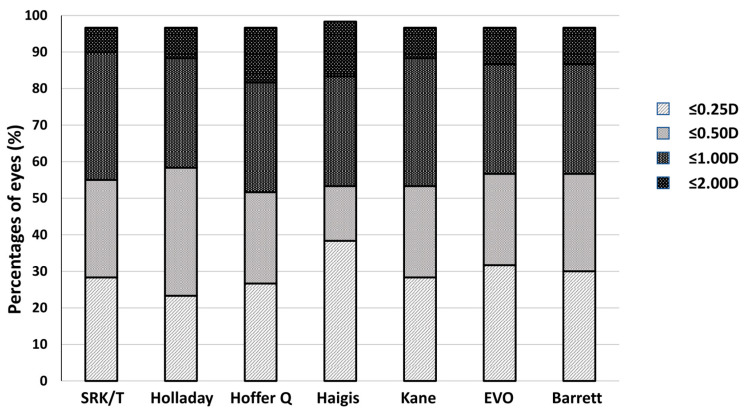
Stacked histograms comparing percentage of eyes within ±0.25 D, ±0.50 D, ±1.00 D and ±2.00 D of predicted spherical equivalent refraction.

The percentage of eyes with prediction errors within ±0.25 D, ±0.50 D, ±1.00 D and ±2.00 D for each formula is reported in Figure 2. At 12 months, the highest percentage of eyes within ±0.50 D was obtained with Holladay 1 (n = 35; 58.3%), Barrett Universal II and EVO (n = 34; 56.7%) and SRK/T (n = 33; 55%). The highest percentage of eyes within ±1.00 D was obtained with SRK/T (n = 54; 90%), Holladay 1 and Kane (n = 53; 88.3%) and Barrett Universal II and EVO (n = 52; 86.7%). However, Fisher’s exact test did not reveal significant differences among the formulas (*p* > 0.05).

**Figure 3:**
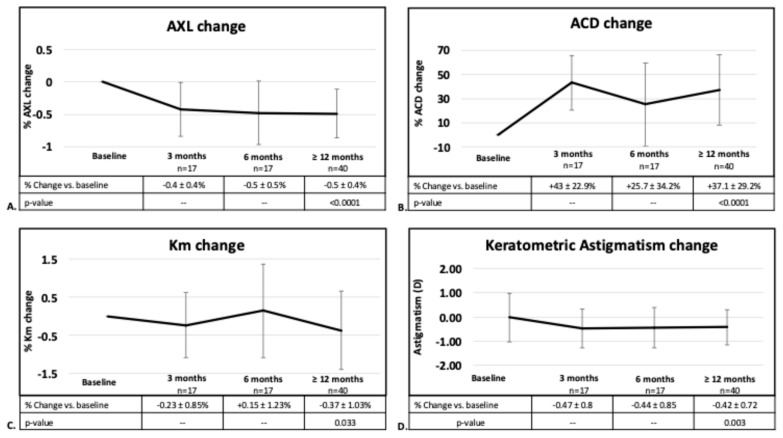
Percentage of change in (**A**) axial length; (**B**) anterior chamber depth; (**C**) mean keratometry; and (**D**) change in keratometric astigmatism at 3 (n = 17 eyes), 6 (n = 17 eyes) and ≥12 months (n = 40 eyes). AXL: axial length; ACD: anterior chamber depth; Km: mean keratometry.

Data on keratometry, ACD and AXL after surgery were available for 17 eyes at 3 and 6 months, and for 40 eyes at ≥12 months. Therefore, descriptive analyses were performed for the 3- and 6-month time points, while formal statistical analysis was limited to the comparison between baseline and ≥12-month data. Compared with preoperative values, AXL significantly shortened, ACD significantly deepened, mean keratometry significantly decreased and keratometric astigmatism significantly changed at ≥12 months. Among the eyes with ≥12-month biometric data, releasable suture removal was performed in 17 eyes (42.5%). No significant differences in Km or keratometric astigmatism change were observed between the eyes that underwent suture removal and those that did not (*p* = 0.498 and *p* = 0.882, respectively) (Figure 3).

In exploratory formula-specific analyses, no consistent association was found between preoperative keratometry, ACD, AXL, and PE across formulas, except for Hoffer Q, for which the PE was positively associated with preoperative ACD (β = 0.55; *p* = 0.020). The PE was positively associated with the percentage of IOP change and the percentage of change in flat keratometry (K1) at ≥12 months for all formulas (all *p* < 0.05). The AE showed significant negative associations with preoperative steep keratometry for the Haigis and Kane formulas. Additionally, the AE for the SRK/T, Holladay 1, Hoffer Q, Haigis, and Kane formulas was associated with the percentage of change in flat keratometry (K1) (*p* < 0.05), whereas the AE for EVO was associated with the percentage of change in steep keratometry (K2) (*p* < 0.05).

## 4. Discussion

This present study evaluated the accuracy of the third-, fourth- and new-generation IOL power calculation formulas in 60 eyes from 40 patients in which combined glaucoma and cataract surgeries were performed. Overall, all the formulas were associated with clinically acceptable refractive outcomes. The PE differed significantly among formulas, with Haigis showing a negative PE and most other formulas showing a tendency toward a hyperopic shift. Accuracy, as measured by estimated marginal mean AE, was similar across formulas, with values ranging from 0.548 to 0.617 D.

Some discrepancies in the direction of the PE shift following phacotrabeculectomy are found in the literature. Hyperopic shifts were detected by Law et al. at approximately 7 months after surgery with the use of an average IOL power obtained from the SRK/T, Holladay, and Hoffer Q [15]; by Chan et al. [17] at about 1 year after surgery using the SRK II formula and by Vaidenau et al., who found a refractive outcome more hyperopic than prediction of 0.53 D within 6 months after surgery [24]. In contrast, myopic shifts were reported in the work from Shin et al. at 1 month after surgery, from Bae et al. at 2 months after surgery, as well as from Tzu et al., who found a myopic shift of 0.5 D compared to phacoemulsification only in a follow-up time shorter than 6 months [13,16,25]. Furthermore, Ong et al. also found a negative PE within 1 year after surgery with, however, no exact data on the time of the refraction measurements and mostly including Chinese subjects [26].

A potential explanation for such discrepancies might lie in the time of the refraction measurements. Works showing myopic PE shifts often collected data within 6 months after surgery, while hyperopic PE shifts appeared to be more frequent with longer follow-up times. In support of this hypothesis, when Chung et al. evaluated the refractive outcomes of phacotrabeculectomy over time, myopic shifts resulted in being more frequent within the first three months after surgery, with a tendency to become hyperopic shifts at 6 months, and to then stabilize at a reasonably good refractive outcome at 1 year after surgery [18]. Such mild increment in the hyperopic shifts was also noticed in the slope of the PE change over time in our study population, however, with most formulas showing hyperopic mean PE already at 3 months after surgery. In this present analysis, only Barrett Universal II showed a significantly positive PE at ≥12 months, whereas Haigis showed a significantly negative PE. Other possible explanations for the discrepancies may rely on AXL measurement techniques and inclusion criteria. Previous studies variably employed PCI, contact A-scan, or immersion A-scan biometry for AXL measurements [13,15,17,18,26], while in post-trabeculectomy eyes noncontact biometry should be preferred [14]. Also, mixed populations, including subjects with different types of glaucoma, may pose a bias due to the expected non-homogeneous anatomical changes following surgery [12,16,19].

Few studies, so far, compared the performance of different IOL power calculation formulas in phacotrabeculectomy. Bae et al. found no significant intergroup differences in AE comparing SRK II, SRK/T, Holladay and Hoffer Q formulas [13]. Although the estimated marginal mean AE was numerically lower with the EVO and Barrett Universal II formulas in our study, we did not find significant differences in the AE among all the tested formulas at ≥12 months postoperatively. However, the overall difference in the PE among formulas was significant, indicating that the absence of significant AE differences should not be interpreted as an absence of inter-formula differences. Rather, formulas appeared to differ mainly in the direction of refractive error, with most formulas showing a hyperopic tendency and Haigis showing a myopic shift, rather than in their overall absolute accuracy. Therefore, despite numerical differences among the formulas, none of the tested formulas showed a clear superiority in terms of absolute prediction accuracy at ≥12 months. However, we measured the refraction at a later stage and only included OAG patients. It is conceivable that the inclusion of angle closure and pseudoexfoliative glaucoma in previous studies might account for different postoperative anatomic changes and, thus, different results [12,19].

To note, the lens thickness (LT) was not measured in our study, since the biometry device used did not report its value. This parameter, incorporated in modern IOL formulas such as Barrett Universal II, Kane, and EVO, potentially improves the prediction of the IOL position, which represents the supposed main source of error in IOL power calculation [27]. In the absence of LT data, modern IOL formulas must rely on internally estimated values or default assumptions, which may reduce their predictive accuracy. Controversies are found in the literature regarding the additional value of LT in the refractive prediction, with a comparative study evaluating the Barrett Universal II, Kane, and EVO formulas, with and without LT as an input, finding that the inclusion of LT did not significantly improve the refractive accuracy of any of these formulas [28] while a subsequent study reached the opposite results [29]. While the lack of LT data in our study may limit the generalizability of the results to high-end surgical settings where full biometric parameters are available, it more accurately reflects real-world clinical scenarios in which biometry devices do not routinely measure the LT.

Another finding from our study showed refractive outcomes at 12 months to be within ±1.00 D of the target in 81.7% to 90% of the eyes. Although being slightly less accurate than in virgin eyes, where more than 90% of the refractive outcomes fall within ±1.00 D of the prediction, the results at 1 year were better compared to data in the literature [30,31]. Previous studies including a phacoemulsification-only control group often showed refractive errors being more frequent after phacotrabeculectomy, however, with a difference frequently not significant [15,17,18,24]. Some of the studies showing significant differences variably employed PCI, contact A-scan, or immersion A-scan biometry for AXL measurements and this might have impacted refractive results [13,25,26].

Anatomical changes, which are known to occur after glaucoma surgery were also evident in our study population [11,12,14,15,32,33,34]. Significant AXL reduction, AC deepening, mean keratometry reduction, and changes in keratometric astigmatism were detected after surgery. No significant differences in Km or keratometric astigmatism change were observed between the eyes that underwent releasable suture removal and those that did not, suggesting that the suture removal was unlikely to be the main driver of the observed keratometric changes. In exploratory formula-specific analyses, PE was consistently associated with the percentage of IOP change and with the percentage change in flat keratometry (K1), whereas associations between the AE and biometric parameters were less uniform across formulas. These findings suggest that postoperative anatomical changes may influence the direction of refractive error more consistently than absolute prediction accuracy. However, the relationship between biometric changes and refractive outcomes after phacotrabeculectomy remains complex and may be partially compensatory. Moreover, we cannot exclude the possibility that the lack of LT data limited our ability to detect subtler interactions between these anatomical parameters and formula accuracy. It is important to underline that only patients with POAG were enrolled, as there is evidence in the literature showing that the type of glaucoma can make a difference in refractive outcome, in particular due to the preoperative ACD values. For example, Shin et al. found that shallower ACD was associated with hyperopic shift and Tabatabaei et al. showed that in some extreme situations like patients with phacomorphic glaucoma, lower generation formulas may still produce more acceptable refractive outcomes [16,35].

We acknowledge several limitations in our study. First, its retrospective design limited the availability of postoperative biometry and LT data. Second, the relatively small sample size may have reduced our ability to detect minor differences in AE. However, a power analysis confirmed that the sample was adequate to detect mean AE differences of 0.25 D between formulas, differences that are clinically relevant in postoperative refractive outcomes. Moreover, our sample size was larger than that of most comparable studies in the literature. Additionally, a control group undergoing phacoemulsification alone was not included. Nonetheless, the literature provides extensive data on refractive outcomes following standard cataract surgery [30,36]. Our study focused exclusively on patients who underwent two-site phacotrabeculectomy; thus, the results may not be generalizable to one-site procedures. Also, we cannot fully exclude the influence of postoperative maneuvers, such as digital massage or releasable suture removal on refractive outcomes. The role of scleral flap sutures in astigmatism induction has been discussed in the literature, with over-tight scleral flap sutures suggested to induce local tissue compression and steepening in the meridian of surgery [37]. However, available studies specifically evaluating suture lysis have not found a significant astigmatic difference between eyes undergoing suture lysis and those without suture lysis [38,39]. In our cohort, bleb needling was not performed, and no significant differences in Km or keratometric astigmatism change were found between eyes that underwent releasable suture removal and those that did not. Finally, although all the IOLs used were one-piece acrylic, variability in the IOL types may have influenced the performance of different formulas in each IOL type subgroup.

## 5. Conclusions

In conclusion, although anatomical changes in the eye following phacotrabeculectomy persist in the long term, all tested formulas demonstrated acceptable prediction accuracy with a substantial proportion of eyes achieving a 12-month refraction within ±1.00 D of target. No significant differences in absolute prediction accuracy were detected among tested formulas; however, the PE differed significantly among formulas. Most formulas showed a tendency toward a hyperopic shift, whereas Haigis showed a myopic shift. This inter-formula difference in the direction of the PE should be considered when selecting the refractive target. The observed hyperopic tendency may be related to postoperative biometric changes, including AXL shortening, AC deepening and mean keratometry reduction, although the relationship between the anatomical changes and refractive prediction error remains complex. Therefore, when using formulas that tend toward hyperopic PE, selecting a slightly myopic refractive target may be a prudent strategy to minimize the risk of postoperative hyperopic surprise.

## Figures and Tables

**Table 1:** Demographics and clinical characteristics of the patient cohort.

Characteristics	Value
Age (years)	71.2 ± 6
Sex (M/F)	29/31
CDVA (LogMAR)	0.3 ± 0.26
IOP (mmHg)	20.6 ± 7.7
MD (dB)	−17.5 ± 8.4
AXL (mm)	23.44 ± 0.88
ACD (mm)	3.1 ± 0.4
Keratometry (steep) (D)	44.5 ± 1.5
Keratometry (flat) (D)	43.3 ± 1.6
Keratometry (mean) (D)	43.9 ± 1.5

CDVA = corrected distance visual acuity; IOP = intraocular pressure; MD = mean deviation; AXL = axial length; ACD = anterior chamber depth; D = diopters.

**Table 2:** Estimated marginal means and 95% confidence intervals derived from linear mixed-effects models for PE and AE. Formulas are sorted by estimated marginal mean AE.

Formula	Estimated Mean PE	95% CI	Estimated Mean AE	95% CI
EVO	0.163	−0.025 to 0.350	0.548	0.398–0.698
Barrett Universal II	0.195	0.008 to 0.383	0.551	0.401–0.701
Holladay 1	0.134	−0.054 to 0.322	0.561	0.411–0.710
SRK/T	0.135	−0.053 to 0.323	0.561	0.411–0.710
Haigis	−0.220	−0.407 to −0.032	0.572	0.422–0.721
Kane	0.034	−0.154 to 0.221	0.577	0.427–0.726
Hoffer Q	0.115	−0.073 to 0.302	0.617	0.467–0.766

AE = absolute error; PE = prediction error; CI = confidence interval.

## Data Availability

Data are available from the corresponding author upon reasonable request.

## References

[1] Spaeth G.L. (2021). European Glaucoma Society Terminology and Guidelines for Glaucoma, 5th Edition. Br. J. Ophthalmol..

[2] Congdon N., O’Colmain B., Klaver C.C.W., Klein R., Muñoz B., Friedman D.S., Kempen J., Taylor H.R., Mitchell P. (2004). Eye Diseases Prevalence Research Group. Causes and prevalence of visual impairment among adults in the United States. Arch. Ophthalmol..

[3] Li S.-W., Chen Y., Wu Q., Lu B., Wang W.-Q., Fang J. (2015). Angle parameter changes of phacoemulsification and combined phacotrabeculectomy for acute primary angle closure. Int. J. Ophthalmol..

[4] El Sayed Y.M., Elhusseiny A.M., Albalkini A.S., El Sheikh R.H., Osman M.A. (2019). Mitomycin C-augmented Phacotrabeculectomy Versus Phacoemulsification in Primary Angle-closure Glaucoma: A Randomized Controlled Study. J. Glaucoma.

[5] Chen P.P., Lin S.C., Junk A.K., Radhakrishnan S., Singh K., Chen T.C. (2015). The Effect of Phacoemulsification on Intraocular Pressure in Glaucoma Patients: A Report by the American Academy of Ophthalmology. Ophthalmology.

[6] Shingleton B.J., Pasternack J.J., Hung J.W., O’Donoghue M.W. (2006). Three and five year changes in intraocular pressures after clear corneal phacoemulsification in open angle glaucoma patients, glaucoma suspects, and normal patients. J. Glaucoma.

[7] Young C.E.C., Seibold L.K., Kahook M.Y. (2020). Cataract surgery and intraocular pressure in glaucoma. Curr. Opin. Ophthalmol..

[8] Wang F., Wu Z.-H. (2016). Phacoemulsification versus combined phacotrabeculectomy in the treatment of primary angle-closure glaucoma with cataract: A Meta-analysis. Int. J. Ophthalmol..

[9] Sacchi M., Monsellato G., Villani E., Lizzio R.A.U., Cremonesi E., Luccarelli S., Nucci P. (2022). Intraocular pressure control after combined phacotrabeculectomy versus trabeculectomy alone. Eur. J. Ophthalmol..

[10] Winuntamalakul Y., Chansangpetch S., Ratanawongphaibul K., Itthipanichpong R., Manassakorn A., Tantisevi V., Rojanapongpun P. (2023). Two-Year Outcomes of Trabeculectomy and Phacotrabeculectomy in Primary Open Angle Versus Primary Angle Closure Glaucoma. J. Glaucoma.

[11] Cashwell L.F., Martin C.A. (1999). Axial length decrease accompanying successful glaucoma filtration surgery. Ophthalmology.

[12] Husain R., Li W., Gazzard G., Foster P.J., Chew P.T., Oen F.T., Phillips R., Khaw P.T., Seah S.K., Aung T. (2013). Longitudinal changes in anterior chamber depth and axial length in Asian subjects after trabeculectomy surgery. Br. J. Ophthalmol..

[13] Bae H.W., Lee Y.H., Kim D.W., Lee T., Hong S., Seong G.J., Kim C.Y. (2016). Effect of trabeculectomy on the accuracy of intraocular lens calculations in patients with open-angle glaucoma. Clin. Exp. Ophthalmol..

[14] Pakravan M., Alvani A., Esfandiari H., Ghahari E., Yaseri M. (2017). Post-trabeculectomy ocular biometric changes. Clin. Exp. Optom..

[15] Law S.K., Mansury A.M., Vasudev D., Caprioli J. (2005). Effects of combined cataract surgery and trabeculectomy with mitomycin C on ocular dimensions. Br. J. Ophthalmol..

[16] Shin J.H., Kim S.H., Oh S., Lee K.M. (2023). Factors Associated with Refractive Prediction Error after Phacotrabeculectomy. J. Clin. Med..

[17] Chan J.C.H., Lai J.S.M., Tham C.C.Y. (2006). Comparison of postoperative refractive outcome in phacotrabeculectomy and phacoemulsification with posterior chamber intraocular lens implantation. J. Glaucoma.

[18] Chung J.K., Wi J.M., Lee K.B., Ahn B.H., Hwang Y.H., Kim M., Jung J.J., Yoo Y.C. (2018). Long-term comparison of postoperative refractive outcomes between phacotrabeculectomy and phacoemulsification. J. Cataract Refract. Surg..

[19] Kader M.A., Pradhan A., Shukla A.G., Maheswari D., Ramakrishnan R., Midya D. (2022). Lowering of intraocular pressure after phacoemulsification in primary open-angle and angle-closure glaucoma: Correlation with lens thickness. Indian J. Ophthalmol..

[20] Savini G., Taroni L., Hoffer K.J. (2020). Recent developments in intraocular lens power calculation methods-update 2020. Ann. Transl. Med..

[21] Holladay J.T., Wilcox R.R., Koch D.D., Wang L. (2021). Review and recommendations for univariate statistical analysis of spherical equivalent prediction error for IOL power calculations. J. Cataract Refract. Surg..

[22] Hoffer K.J., Savini G. (2021). Update on Intraocular Lens Power Calculation Study Protocols: The Better Way to Design and Report Clinical Trials. Ophthalmology.

[23] Kang Y.S., Sung M.S., Heo H., Ji Y.S., Park S.W. (2021). Long-term outcomes of prediction error after combined phacoemulsification and trabeculectomy in glaucoma patients. BMC Ophthalmol..

[24] Vaideanu D., Mandal K., Hildreth A., Fraser S.G., Phelan P.S. (2008). Visual and refractive outcome of one-site phacotrabeculectomy compared with temporal approach phacoemulsification. Clin. Ophthalmol..

[25] Tzu J.H., Shah C.T., Galor A., Junk A.K., Sastry A., Wellik S.R. (2015). Refractive outcomes of combined cataract and glaucoma surgery. J. Glaucoma.

[26] Ong C., Nongpiur M., Peter L., Perera S.A. (2016). Combined Approach to Phacoemulsification and Trabeculectomy Results in Less Ideal Refractive Outcomes Compared with the Sequential Approach. J. Glaucoma.

[27] Norrby S. (2008). Sources of error in intraocular lens power calculation. J. Cataract Refract. Surg..

[28] Taroni L., Hoffer K.J., Lupardi E., Barboni P., Savini G. (2021). Accuracy of New Intraocular Lens Power Calculation Formulas: A Lens Thickness Study. J. Refract. Surg..

[29] Wendelstein J.A., Rothbächer J., Heath M., McDonald M.C., Hoffmann P.C., Cooke D.L., Seiler T.G., Langenbucher A., Riaz K.M. (2023). Influence and predictive value of optional parameters in new-generation intraocular lens formulas. J. Cataract Refract. Surg..

[30] Kane J.X., Van Heerden A., Atik A., Petsoglou C. (2016). Intraocular lens power formula accuracy: Comparison of 7 formulas. J. Cataract Refract. Surg..

[31] Melles R.B., Holladay J.T., Chang W.J. (2018). Accuracy of Intraocular Lens Calculation Formulas. Ophthalmology.

[32] Martínez-Belló C., Rodriguez-Ares T., Pazos B., Capeáns C., Sánchez-Salorio M. (2000). Changes in anterior chamber depth and angle width after filtration surgery: A quantitative study using ultrasound biomicroscopy. J. Glaucoma.

[33] Vernon S.A., Zambarakji H.J., Potgieter F., Evans J., Chell P.B. (1999). Topographic and keratometric astigmatism up to 1 year following small flap trabeculectomy (microtrabeculectomy). Br. J. Ophthalmol..

[34] Egrilmez S., Ates H., Nalcaci S., Andac K., Yagci A. (2004). Surgically induced corneal refractive change following glaucoma surgery: Nonpenetrating trabecular surgeries versus trabeculectomy. J. Cataract Refract. Surg..

[35] Tabatabaei S.A., Samadi M., Soleimani M., Fonoodi H., Ghods S., Inanloo B. (2022). Accuracy of different lens power calculation formulas in patients with phacomorphic glaucoma. Taiwan. J. Ophthalmol..

[36] Khoramnia R., Auffarth G., Łabuz G., Pettit G., Suryakumar R. (2022). Refractive Outcomes after Cataract Surgery. Diagnostics.

[37] Dietze P.J., Oram O., Kohnen T., Feldman R.M., Koch D.D., Gross R.L. (1997). Visual function following trabeculectomy: Effect on corneal topography and contrast sensitivity. J. Glaucoma.

[38] Rosen W.J., Mannis M.J., Brandt J.D. (1992). The effect of trabeculectomy on corneal topography. Ophthalmic Surg..

[39] Delbeke H., Stalmans I., Vandewalle E., Zeyen T. (2016). The Effect of Trabeculectomy on Astigmatism. J. Glaucoma.

